# Pseudochamber-Protected Keratoplasty (PPK) with a New Inter-Corneal Surgical Device Implant Technique in High-Risk Cases

**DOI:** 10.3390/jcm13195715

**Published:** 2024-09-25

**Authors:** José F. Alfonso, Carlos Lisa, Belén Alfonso-Bartolozzi, Rosa Alvarado-Villacorta, David Madrid-Costa, Luis Fernández-Vega-Cueto

**Affiliations:** 1 Fernández-Vega Ophthalmological Institute (Instituto Oftalmológico Fernández-Vega), Avda. Dres. Fernández-Vega 114, 33012 Oviedo, Spain; carloslisa@fernandez-vega.com (C.L.); belen.alfonso@fernandez-vega.com (B.A.-B.); rosa.alvarado.v@upch.pe (R.A.-V.); lfvc@fernandez-vega.com (L.F.-V.-C.); 2 Clinical and Experimental Eye Research Group (CEER), Optometry and Vision Department, Faculty of Optics and Optometry, Universidad Complutense de Madrid, 28040 Madrid, Spain; damadrid@ucm.es

**Keywords:** corneal transplant, pseudochamber-protected keratoplasty (PPK), Endo-K Pro^®^ implant

## Abstract

**Background/Objectives**: To describe the pseudochamber-protected keratoplasty (PPK) procedure with the new Endo-K Pro^®^ implant technique and report the clinical outcomes in patients at high risk for penetrating keratoplasty (PKP). **Methods**: This case series study included patients who required a PKP and had a high risk for corneal transplant failure. All cases underwent the PPK procedure with simultaneous Endo-K Pro^®^ implantation and had a minimum follow-up of 12 months. Graft survival was the primary outcome (defined as a clear graft with an endothelial cell density >500 cells/mm^2^). Central corneal thickness (CCT), corrected distance visual acuity (CDVA), intraocular pressure (IOP), and complication rate were the secondary outcomes. **Results**: Twenty-five eyes (twenty-five patients) were included. The mean follow-up was 23.64 ± 8.2 months (range: 12–36 months). Graft survival was achieved in 23 of the 25 cases (92%). One eye had to be re-transplanted due to persistent oedema secondary to uncontrolled IOP. In two cases (8%), the graft failed three months after surgery when an anterior pseudochamber collapsed due to direct contact of donor endothelium and host tissue. Six eyes experienced host tissue protrusion that was successfully managed using an Nd: YAG laser (two eyes) or injecting a cohesive viscoelastic into the pseudochamber (four eyes). CDVA increased significantly during the follow-up period. No significant changes were found in IOP. No intra- or postoperative complications were reported. **Conclusions**: PPK with the Endo-K Pro^®^ implant seems to be an effective and safe surgical approach as an alternative in high-risk patients for PKP, allowing full-thickness corneal transplantation without performing an open-sky procedure.

## 1. Introduction

Nowadays, penetrating keratoplasty (PKP) is being replaced by lamellar keratoplasty procedures. The potential postoperative complications of PKP and the development of new surgical devices have led to the emergence of and a rise in lamellar surgery [1,2,3,4,5,6,7,8]. However, surgeons often face diseases that either affect the entire cornea or where the aetiology or severity of the underlying disease disadvises lamellar keratoplasties and, hence, where a PKP must be recommended. Therefore, even though lamellar procedures are imposed, it is interesting to take on the challenge of acquiring new techniques for full-thickness corneal transplantation that boast higher safety rates than PKP and that could even be a surgical option prior to keratoprosthesis or an alternative to it [9].

Lazar [10] and Loewenstein [11] proposed a method for a full-thickness donor corneal graft that maintains the Descemet membrane and endothelium of the host cornea in some selected cases of bullous keratopathy. This approach aimed to increase the safety of the PKP by not opening the anterior chamber (closed-system procedure) and consequently completing the PKP as an extraocular procedure. For this approach to be successful, it is crucial that a layer of aqueous humor separates the host’s Descemet membrane from the graft. Otherwise, if the retained Descemet membrane comes into contact with the graft, the donor’s endothelium will be compromised, leading to graft failure. Despite this, the authors thought that even though this extraocular procedure failed, the odds of success of a later conventional PKP would not be reduced, suggesting that this modified keratoplasty technique could be helpful in high-risk cases [10,11].

Following these attempts, we previously reported a new surgical approach termed “pseudochamber-protected keratoplasty (PPK)” [12,13] based on the lamellar dissection of host tissue up to the pre-Descemetic level, a full-thickness corneal graft suture, and injecting a cohesive viscoelastic to create a space between the donor and recipient corneas. The potential benefits of this surgical approach are, firstly, that it avoids an open-sky procedure, and secondly, the remaining recipient’s tissue acts as a barrier to the donor’s endothelium, thus protecting it from potential complications that might arise inside the eyeball (infection, inflammatory reaction) and from surgical maneuvers in cases requiring a combined intraocular surgery (cataract, vitreoretinal, or glaucoma). Finally, the space between both corneas, defined as the pseudochamber (a term used to distinguish between the artificially made space and the real anterior chamber), prevents contact between the host bed and donor endothelium. While this pseudochamber is preserved, the donor’s endothelium functions appropriately and the graft remains clear; however, as the viscoelastic is removed and exchanged for aqueous humor, the pseudochamber’s space gradually decreases, and consequently, the two corneas move closer together. If the graft and recipient cornea come into contact, the donor’s endothelium viability is compromised, leading to graft failure.

To overcome this issue, a new inter-corneal implant (Endo-K Pro^®^; AJL Ophthalmic, Spain) was designed to maintain the pseudochamber and, in this way, to improve graft survival in high-risk cases, avoiding open-sky procedures. Herein, we aim to report the clinical outcomes of patients who underwent PPK with this new Endo-K Pro^®^ implant.

## 2. Materials and Methods

This case series study included patients who required a PKP and had a high risk for corneal transplant failure. Cases with full-thickness corneal opacity secondary to chemical/thermal or physical trauma or with failed corneal graft were selected. Cases with a previous PKP were excluded. This study was conducted according to the tenets of the Declaration of Helsinki and received the Institutional Ethics Committee’s approval. Written informed consent was obtained from every patient during surgical planning. 

All cases underwent the PPK procedure with simultaneous Endo-K Pro^®^ implantation and had a minimum follow-up of 12 months.

### 2.1. Endo-K Pro^®^ Technical Characteristics

**Figure 1:**
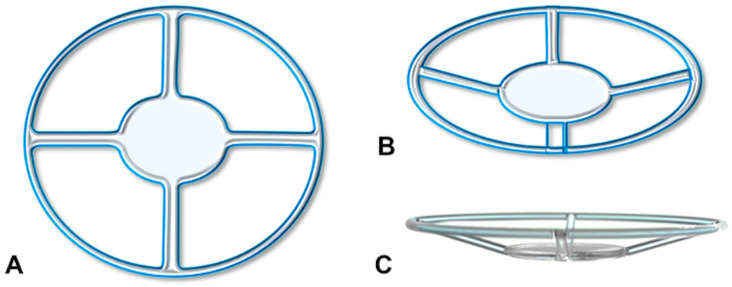
Schematic design of the Endo-K Pro^®^ implant depicting the top (**A**), perspective (**B**), and side view (**C**). The two-level design enables the pushing backwards of the stromal bed of the host cornea far from the donor cornea endothelium.

The Endo-K Pro^®^ implant (AJL Ophthalmic, Vitoria, Spain) is a single-piece device made of medical-grade polymethyl methacrylate. Its design includes an optically neutral 3 mm central disc and four 0.5 mm wide angled spokes that radiate from the disc toward an outer ring, leaving the central disk in a plane below the ring (Figure 1). There are four commercially available ring diameters ranging from 7.50 to 9.00 mm in steps of 0.50 mm. It is worth noting that the Endo-K Pro^®^ implant should be entitled as an inter-corneal implant, not a keratoprosthesis, since it is a device implanted between the donor cornea and the host cornea tissue and does not replace the host cornea.

### 2.2. Surgical Technique

**Figure 2:**
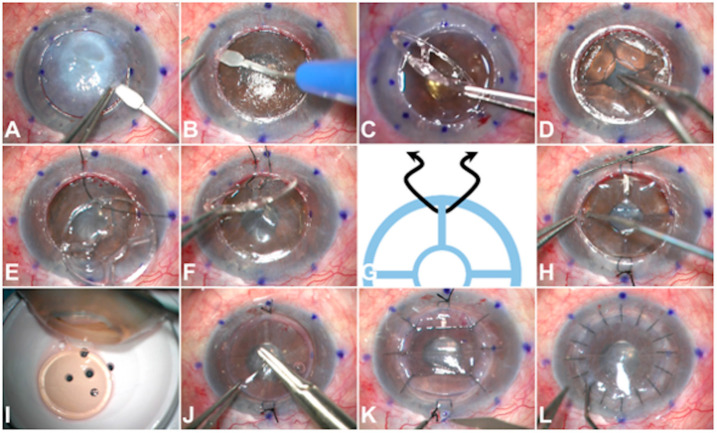
Intraoperative captions showing the surgical steps of the pseudochamber-protected keratoplasty with the Endo-K Pro^®^ implant technique. First, a partial trephination up to 70% thickness was performed, and corneal stroma was carefully dissected by using the manual layer-by-layer technique to obtain a stromal bed thinner than 100 µm (**A**). Then, a 360-degree 0.25 mm wide peripheral pocket was dissected (**B**) to place the Endo-K Pro^®^ implant properly. The outer ring, which was located upward, was carefully taken and inserted into the peripheral pocket (**C**), while the central disk pushed backward the stromal bed of the host cornea (**D**). After that, the Endo-K Pro^®^ implant was provisionally fixed with two double-arm 8-0 silk sutures (**E**). It is recommended that these provisional sutures run under the spoke and over the outer ring (**F**,**G**). Then, the outer ring was properly introduced into the peripheral pocket (**H**). Eventually, the 0.25 mm oversized full-thickness donor button, including the endothelium (**I**), was sutured using interrupted 10-0 monofilament nylon sutures and running just over the outer ring of the Endo-K Pro^®^ implant (**J**). Once the cardinal sutures were in place, the silk sutures were removed (**K**), the graft suturing was completed with 16 interrupted sutures, and the cohesive viscoelastic or balanced salt solution was used to fill the anterior pseudochamber (**L**).

All surgeries were performed by the same experienced surgeon (JFA) under sedation and regional anaesthesia at Fernandez-Vega University Institute. The surgical steps are depicted in Figure 2 and Supplementary Material digital content Video S1. The technique can be divided into three steps as follows:

*Host cornea dissection*

First, the host cornea was marked to center the trephine blade (Barron Radial Vacuum Trephine, Kathena©, Grand Blanc, MI, USA), and trephination was performed to a depth of 70% thickness. Then, the corneal stroma was carefully dissected by using the manual layer-by-layer technique (described by Anwar) [14] (Figure 2A) to obtain a stromal bed thinner than 100 µm (preferably between 50 and 75 µm). The residual stromal bed thickness was intraoperatively verified by real-time optical coherence tomography (OCT) (OPMI LUMERA 700, Zeiss©, Zeiss, Oberkochen, Germany) and ultrasound pachymetry (SonoGage, Cleveland, OH, USA).

*Endo-K Pro® implantation*

To properly place the Endo-K Pro^®^ implant, a 360-degree 0.25 mm wide peripheral pre-Descemetic pocket was created (Figure 2B). In other terms, if the host cornea trephination was 8 mm in diameter, an 8.5 mm diameter pocket dissection was performed. After that, the Endo-K Pro^®^ implant was inserted into the pocket so that the central disk pushed the residual bed of the host cornea backwards (Figure 2C,D). At this point, the Endo-K Pro^®^ implant was fixed to the host peripheral cornea with two provisional double-arm 8-0 silk sutures to facilitate the next step (Figure 2E). It is important to note that these provisional sutures must run under the spoke and over the outer ring of the Endo-K Pro^®^ implant (Figure 2F,G), allowing the outer ring to be appropriately inserted into the peripheral pocket (Figure 2H).

*Donor cornea*

Finally, the 0.25 mm oversized full-thickness donor button, including the endothelium (Figure 2I), was sutured using interrupted 10-0 monofilament nylon sutures and running just over the outer ring of the Endo-K Pro^®^ implant (Figure 2J). Once the cardinal sutures were in place, the provisional silk sutures were removed (Figure 2K), and the graft suturing was completed with conventional 16 interrupted 10/0 nylon sutures. A cohesive viscoelastic or balanced salt solution (BSS) was injected to fill the newly created pseudochamber (Figure 2L).

### 2.3. Outcomes Measurements and Postoperative Care

All patients underwent comprehensive ophthalmologic examination, including slit-lamp examination, tonometry, non-contact specular microscopy (SP 2000P, Topcon^®^ Europe, Capelle a/d IJssel, the Netherlands), and anterior segment optical coherence tomography (AS-OCT) (Casia SS-OCT v.2, Tomey^®^, Nagoya, Japan). Graft survival was the primary outcome and was defined as a clear graft with an endothelial cell density (ECD) higher than 500 cells/mm^2^. Central corneal thickness (CCT), corrected distance visual acuity (CDVA), intraocular pressure (IOP), additional procedures needed, and complication rate were the secondary outcomes. IOP measurement was performed using a rebound tonometer (iCare^®^ IC200, Icare Tiolat Oy, Helsinki, Finland).

The postoperative protocol to assess the graft survival, implant tolerance, and surgery viability was as follows: (1) slit lamp examination to assess graft clarity and implant tolerance and monitor the success of the pseudochamber maintenance over time; (2) AS-OCT to measure the corneal thickness and monitor the distance of the central disk from the donor cornea endothelium (pseudochamber depth) and the 360° peripheral contact between the donor and host corneas; (3) non-contact specular microscopy to assess the donor endothelium health over time (being unable to perform this assessment due to significant cornea oedema was considered a sign of graft failure); and (4) IOP control to evaluate the prognosis and the need for subsequent surgeries. 

### 2.4. Postoperative Management of the Residual Stromal Bed

**Figure 3:**
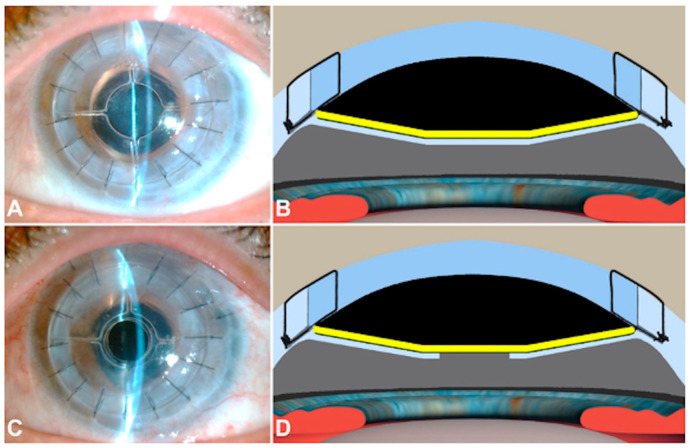
Residual stromal bed management. The first option is to leave it in place in cases with poor visual prognosis (**A**,**B**). The second option is to remove only the residual bed that involves the visual axis (**C**,**D**). It should be noted that despite opening the stromal bed, the optically neutral central disk of the Endo-K Pro^®^ implant (drawn as a yellow line) keeps the pseudochamber closed.

Regarding postoperative management of the stromal bed, two approaches can arise during the follow-up depending on the visual prognosis: preserve the residual host tissue in cases where the visual prognosis is poor or the visual acuity is unlikely to improve upon removal (Figure 3A,B); or in patients with a potential visual gain, perform a central hole in the host tissue just below the central disk of the Endo-K Pro^®^ implant to open the occluded visual axis (Figure 3C,D). 

**Figure 4:**
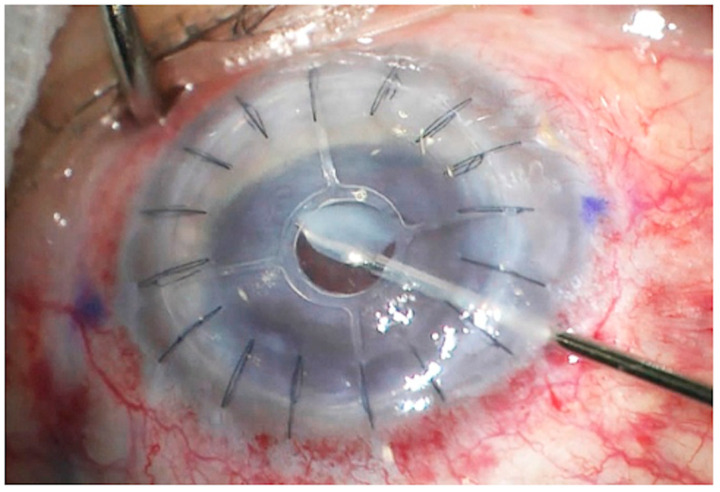
Intraoperative caption showing the host tissue removal using a 20 G vitrectomy scissor. Note that the entry point is at the limbus level, reaching the posterior surface of the host tissue.

To this extent, the host tissue was removed with Nd: YAG if the residual bed host tissue was thinner than 80 µm or with a femtosecond laser or with vitrectomy scissors through a limbus incision (Figure 4) is the residual bed thickness was thicker than 80 µm. This procedure was performed at least six months after surgery when the graft was clear enough and donor–host interface wound healing was achieved.

### 2.5. Statistical Analysis

Visual inspection with Q-Q plots and the Shapiro–Wilk test were used to assess the normality of quantitative data. CDVA values were converted to logarithms of the minimum angle of resolution (LogMAR). Descriptive statistical data were expressed as means ± standard deviation (SD) whether they had normal distribution; otherwise, the median and interquartile range (IQR) were used. The categorical variables were expressed as numbers (n) and percentages (%). Comparisons between preoperative and postoperative values were analyzed using the Wilcoxon signed-rank test using the Stata^®^ software version 15.1 (StataCorp. 2015, Stata Statistical Software: Release 15. College Station, TX, USA: StataCorp LP.).

## 3. Results

This study included 25 eyes of 25 patients with a mean age of 67.5 ± 12.9 (range 36 to 86). The mean postoperative follow-up was 23.64 ± 8.2 (range from 12 to 36 months). Table 1 summarizes the patients’ demographic and preoperative data. A total of 13 eyes (52%) had a history of failed lamellar keratoplasty (in 5 cases, an anterior lamellar keratoplasty, and in 8 cases, posterior lamellar keratoplasty).

**Figure 5:**
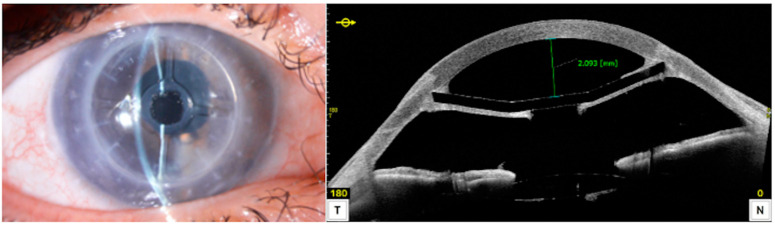
A representative case showing the 24-month slit lap view (**left**), a cross-sectional image obtained by anterior segment optical coherence tomography (**right**).

Graft survival (defined as a clear graft with an endothelial cell density (ECD) higher than 500 cells/mm^2^) was achieved in 23 of the 25 cases (92%). Figure 5 shows a successful case at 24 months postoperatively. 

**Figure 6:**
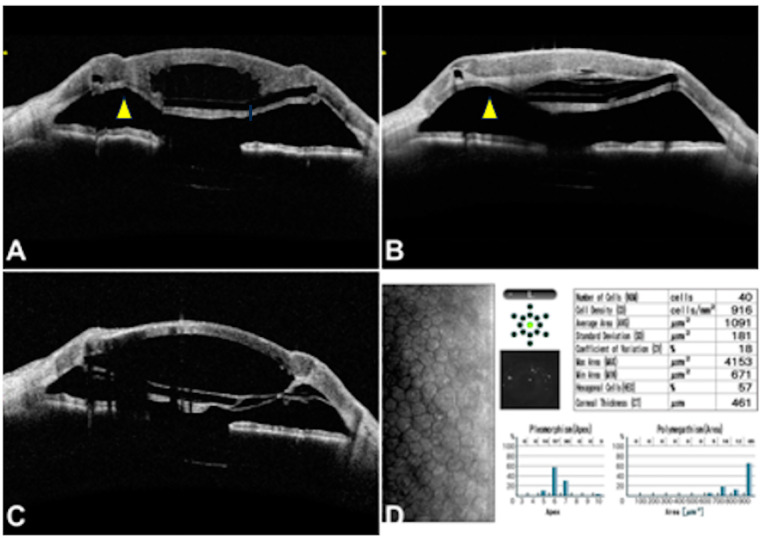
This 69-year-old male glaucoma patient had persistent ocular hypertension in the early postoperative period, leading to a significant host tissue protrusion (yellow arrowhead) that arose through the space between the spokes of the implant (**A**), which came in contact with the donor endothelium and caused the detachment of the Descemet membrane, leading to irreversible corneal decompensation (**B**). After that, a second graft was performed once the IOP was controlled. Intraoperatively, the stromal bed was dissected slightly to make it thinner, and the peripheral pocket dissection was checked while keeping the same implant. Two years later, the new graft remained clear, maintaining the pseudochamber’s depth (**C**) and a good endothelial cell density (**D**).

It is noteworthy that one of these successful cases had to be re-transplanted at 15 months due to persistent oedema secondary to uncontrolled IOP (Figure 6A–D). Figure 6 depicts the temporal sequence of the events that occurred in this case. The corneal oedema provoked a significant protrusion of the host tissue that arose through the space between the spokes of the Endo-K Pro^®^, which came in contact with the donor endothelium and caused the detachment of its Descemet membrane, leading to irreversible corneal decompensation (Figure 6B). In this case, the first step was to decrease the IOP with medical therapy. Once the IOP was controlled, a new corneal transplant was scheduled. Firstly, intraoperatively, the stromal bed was dissected slightly to make it thinner, and the peripheral pocket dissection was checked while keeping the same implant. Finally, the donor cornea was sutured following the same procedure as in the original PPK technique. Two years later, the new graft remained clear, maintaining the pseudochamber’s depth (Figure 6C) and a good endothelial cell density (Figure 6D). 

**Figure 7:**
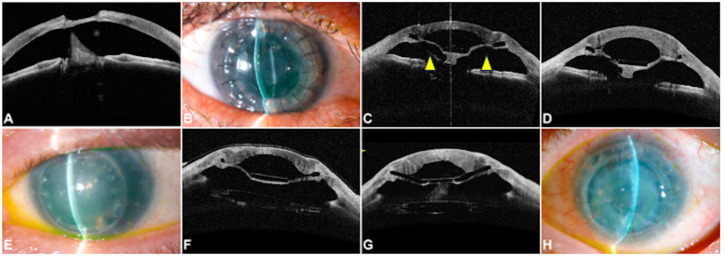
(**A**–**H**) This 59-year-old female patient had a history of recurrent corneal ulcers and advanced cataracts. At the presentation, she had a large stromal defect and signs of cataract reabsorption (**A**). After surgery, the anterior segment reconstruction was achieved (**B**); however, three months postoperatively, the host tissue protruded (yellow arrowhead) (**C**), leading to peripheral contact with the donor endothelium, causing graft failure (**D**) despite attempts to restore the pseudochamber depth by introducing a cohesive viscoelastic. (**E**–**H**) This patient had graft failure after a previous deep anterior lamellar keratoplasty, aniridia, and a posterior intraocular lens preoperatively (**E**). Three months after Endo-K Pro^®^ implantation, a fibrin membrane covering the anterior surface of the device was seen, leading to peripheral contact (**F**,**G**) and, finally, graft failure (**H**).

In two cases (8%), the graft failed three months after surgery due to the anterior pseudochamber collapsing as a consequence of direct contact with the donor endothelium and host tissue. Figure 7 reports the clinical findings and details the patient characteristics in each case. The main related factors in these cases were the protrusion of the host tissue between the spokes, leading to direct contact between the donor and host tissues in the first case (Figure 7A–D) and the fibrin membrane formation covering the anterior surface of the implant, whose contraction induced the peripheral contact between the donor endothelium and the host tissue, in the second one (Figure 7E–H). 

Six months after surgery, the median ECD was 1309 cells/mm^2^ (range: 630–2465 cells/mm^2^), and it did not change significantly over the follow-up (*p* = 0.052). At the last follow-up visit, all eyes had an ECD ≥ 500 cells/mm^2^, and more than half of the cases (13/25, 56.5%) achieved an ECD ≥ 1000 cells/mm^2^. CCT reduced significantly at six months postoperatively and remained unchanged until the last assessment. No significant changes were found in IOP (Table 2).

No intraoperative complications were reported. A proper lamellar dissection and Endo-K Pro^®^ implantation were performed in all cases. During follow-up, there were no extrusion or infection cases. In 16 cases (64%) with potential visual function, the central area of the stromal bed was removed using a femtosecond laser (n = 6), vitrectomy scissors (n = 1), or an Nd: YAG laser (n = 9); in these eyes, CDVA increased significantly during the follow-up period (Table 2).

**Figure 8:**
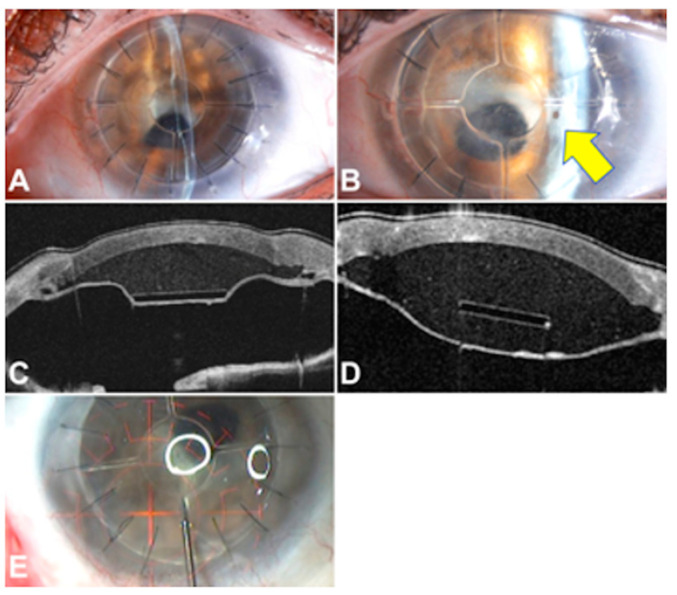
(**A**–**E**) Additional procedures: (**A**) Pre- and (**B**) postoperative clinical photographs of Nd: YAG laser application on host tissue of one case to resolve its protrusion (yellow arrow points to the hole). (**C**) Before and (**D**) after OCT views of cohesive viscoelastic injection. It should be noted that the 30G needle should be inserted through the donor cornea just above one of the spokes to avoid accidental host tissue perforation (**E**).

Regarding postoperative management, six eyes experienced host tissue protrusion that was successfully managed as follows: in two eyes, an Nd: YAG laser was used to puncture the protruded host tissue (Figure 8A,B), and in the other four eyes, a cohesive viscoelastic was injected into the pseudochamber (Figure 8C,D). The cohesive viscoelastic was injected using a 30G needle that had to be inserted through the donor cornea just above one of the spokes to avoid an unintentional perforation of the host tissue (Figure 8E).

**Figure 9:**
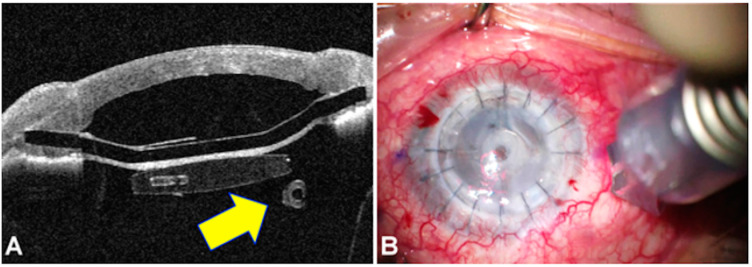
(**A**) Cross-sectional optical coherence tomography view of a patient with a glaucoma drainage device (yellow arrow) implantation to control the intraocular pressure. (**B**) Intraoperative caption of one case where a cyclophotocoagulation procedure was needed.

Two eyes required a secondary surgical procedure for decreasing a high IOP that was not induced by the PPK technique or Endo-K Pro^®^ implant but could have compromised its viability. Specifically, these two cases had a preoperative high IOP. Postoperatively, to maintain the pseudochamber in optimal conditions, avoiding contact between the donor endothelium and the host tissue due to high IOP, one case required a postoperative glaucoma drainage device implantation (Figure 9A) and one required postoperative cyclophotocoagulation (Figure 9B). 

## 4. Discussion

Traditionally, corneal transplantation has been described as the most successful human transplantation surgery, with a success rate that ranges between 75 and 80% at ten years of follow-up in primary corneal grafts placed in avascular ‘low-risk’ beds [15]. However, this survival rate drops to from 30 to 50% at 3 to 5 years of follow-up in cases of ‘high-risk’ beds [16]. These patients have multiple factors, such as inflammation, neovascularization, and limbal stem cell niche disturbances, which lead to the breakdown of the immune privilege of the cornea [17,18]. In the current study, graft survival in these high-risk cases following PPK with the Endo-K Pro^®^ implant was achieved in 23 of the 25 cases (92%) over a postoperative follow-up of 23.64 ± 8.2 months.

Our target population included patients with risk factors of poor prognosis outcome of PKP, such as patients with a previous intraocular surgery (e.g., aphakia, pseudophakia, post glaucoma drainage device implantation, or post vitrectomy with silicone oil) or a history of anterior segment inflammatory disorder with or without synechia, patients with intraocular-pressure-induced endothelial dysfunction with or without limbal stem cell deficiency, cases secondary to chemical/thermal or physical trauma, or cases with a failed corneal graft [16,17] (Table 1). Our clinical outcomes suggest that PPK with the Endo-K Pro^®^ implant might be an alternative approach in these challenging conditions. 

Several strategies have been published, including local and systemic immunosuppression and the adoption of newer surgical techniques for high-risk cases [18]. As such, keratoprosthesis has emerged as an option in so-called end-stage corneal diseases, showing a better prognosis than repeated and primary PKP in high-risk cases [19]. However, since keratoprosthesis still requires a penetrating surgery, there is a lifetime risk of sight-threatening complications, including retroprosthetic membrane formation, sterile keratolysis, infectious keratitis and endophthalmitis, and glaucoma-associated and vitreoretinal complications [9,20]. 

PPK with the Endo-K Pro^®^ implant approach can be defined as a full-thickness corneal transplant performed through a non-penetrating procedure. This technique has two main advantages that may contribute to increasing the graft survival rate in high-risk cases: First, it avoids an open-sky surgery, limiting the likelihood of intra- or postoperative-related complications [1,2,3,4] and allowing combined simultaneous (e.g., lensectomy) or sequential procedures in a closed system, such as glaucoma surgery. Furthermore, repeat corneal transplantation can be safely performed while maintaining the implant in place in cases of graft failure. Second, the pseudochamber provides endothelial cells with a protective microenvironment, especially when the anterior segment is severely disrupted and chronically inflamed, as in our cases, where more than half had iris alterations and a history of failed keratoplasty and where all cases had a previous lens surgery. Endothelial imaging using specular microscopy showed that all eyes had an ECD ≥ 500 cells/mm^2^ at the last follow-up, and more than half of our cases achieved at least 1000 cells/mm^2^. Furthermore, no significant changes in IOP were recorded.

The proposed surgical technique does not require substantial modifications of the conventional anterior lamellar keratoplasty; however, one of the main intraoperative considerations of this new approach is achieving an intact stromal bed thick enough to support the implant. For this purpose, we preferred performing a careful lamellar dissection by using the manual layer-by-layer technique instead of the big-bubble technique, especially for those cases with deep corneal scars, to reduce the risk of accidental perforation of the Descemet membrane [5]. We suggest leaving a stromal bed thickness of between 50 and 100 µm. Thicker beds may limit postoperative visual acuity, while thinner ones may hinder the proper implantation of the device and may also predispose tissue protrusion toward the donor’s endothelium. Of note is the fact that this surgical technique does not significantly increase the complexity compared to the manual layer-by-layer technique. This means that the main complexity is related to manual dissection. Consequently, this procedure should not represent a great challenge for an experienced corneal surgeon using the manual dissection technique. However, this surgery will require a learning curve for an inexperienced cornea surgeon. 

Postoperative care is a paramount part of the success of this method, mainly because close monitoring of the pseudochamber is required to avoid direct contact between the host tissue and the endothelium’s donor. The collapse of this pseudochamber can be prevented and managed by three approaches: First, having a medically or surgically well-controlled IOP before the procedure. Preoperative glaucoma drainage device implantation may be one of the best available options [21]. Second, Nd: YAG laser impacts may be performed on the residual bed to equalize the pressure between the anterior chamber and the pseudochamber. Although this option may be safe and effective in most cases, it is not advisable in vitrectomized eyes filled with silicone oil because this could move into the pseudochamber; and third, in cases where the options mentioned above were ineffective or could not be performed, a cohesive viscoelastic could be injected using a 30 G needle through the donor cornea to reopen the pseudochamber. As the viscoelastic is gradually exchanged with aqueous humor, this procedure may need to be repeated, and it could be performed as long as there is a reasonable chance of success. In our cohort, the protrusion of the stromal bed tissue occurred in six cases, where two were successfully managed with an Nd: YAG laser and four involved viscoelastic injection into the pseudochamber. General advice could be to use an Nd: YAG laser as soon as the peripheral tissue protrusion is detected and before it reaches the donor’s endothelium. On the other hand, when the host stromal bed comes into direct contact with the donor’s endothelium, viscoelastic injection could be the best option to separate these tissues. 

Once the stability of the pseudochamber is achieved and the cornea is clear enough, another postoperative concern is the presence of the residual host stromal bed that becomes more fibrotic over time. In cases with a potential visual function where the visual acuity is likely to improve, we removed the tissue over the visual axis at least six months postoperatively. In eyes with a residual tissue thickness thinner than 80 µm, the tissue was removed through the use of an Nd: YAG laser, while in eyes with a thicker residual stromal bed, a femtosecond laser and/or vitrectomy scissors were required. It should be pointed out that the vitrectomy scissors should be introduced through the limbus, reaching the posterior surface of the stromal bed (i.e., the host endothelium side). Furthermore, a femtosecond laser allows for more accurate and regular tissue cutting. 

Our results should be interpreted considering the study’s limitations. First, we only included data from patients without a previous PKP, which may introduce selection bias. Cases with a failed PKP deserve special consideration due to the presence of a penetrating wound at the host–tissue interface, which may hinder the critical steps of the surgery, such as the lamellar dissection and the 360-degree pocket creation at the pre-Descemetic level, which in turn decreases the likelihood of a satisfactory outcome. Therefore, the clinical outcomes of this new surgical technique on those specific patients are currently the scope of another analysis and will be reported separately. Second, the limited sample size and the lack of a long-term follow-up may not allow us to obtain more accurate information to analyze the comparative effectiveness of different approaches. However, we are focused on patients with poor or uncertain prognoses in terms of PKP, representing only a subgroup of patients in daily practice. On the other hand, this is the first prospective clinical study reporting clinical outcomes of this procedure; thus, these issues should be addressed in future studies. 

We are currently making changes to the original surgical technique by increasing the stromal pocket width. This will allow us to implant an Endo-K Pro^®^ implant with a diameter that is 0.75 mm larger than the host tissue. 

## 5. Conclusions

In summary, PPK with an Endo-K Pro^®^ implant seems to be an effective and safe surgical approach as an alternative in high-risk patients for PK, allowing a full-thickness corneal transplantation without performing an open-sky procedure. Satisfactory outcomes could be achieved if the indication for surgery and follow-up are properly assessed. Further technological developments are being made to improve these initial results.

## Figures and Tables

**Table 1:** Demographic data and preoperative clinical profile (n = 25 patients).

	Value
**Age (years), mean ± SD (range)**	67.5 ± 12.9 (36–86)
**Female/male ratio**	8/17
**Without previous keratoplasty, n (%)**	12 (48.0)
**With previous keratoplasty, n (%)**	13 (52.0)
* DMEK, n (%)*	6 (46.1)
* DALK, n (%)*	5 (38.5)
* DSAEK, n (%)*	2 (15.4)
**IOP (mmHg), median (IQR) [range]**	12 (11–14) [9–31]
**CCT (µm), median (IQR) [range]**	739 (621–847) [547–1662]
**Corneal findings, n (%)**	
* Edema*	24 (96)
* Deep neovascularization*	1 (4)
**Iris findings, n (%)**	
* No alterations*	11 (44)
* Sinechyiae*	5 (20)
* Areflexic mydriasis*	3 (12)
* Aniridia*	3 (12)
* Pseudoexfoliation*	2 (8)
* Atrophy*	1 (4)
**Lens surgery, n (%)**	
* Aphakia*	4 (16)
* Posterior chamber IOL*	15 (60)
* Iris-claw IOL*	3 (12)
* Anterior chamber IOL*	2 (8)
* Scleral-fixated IOL*	1 (4)
**IOP control, n (%)**	
* No treatment*	15 (60)
* Medical therapy alone*	4 (16)
* Medical therapy + Glaucoma drainage device*	3 (12)
* Medical therapy + Filtering surgery*	1 (4)
* Medical therapy + CFC*	1 (4)
* CFC alone*	1 (4)
**Retina findings, n (%)**	
* No alterations*	21 (84)
* Previous retinal detachment*	3 (12)
* Macular edema*	1 (4)
**Vitreous cavity, n (%)**	
* No alterations*	19 (76)
* Vitrectomy surgery without silicon oil*	5 (20)
* Vitrectomy surgery with silicon oil*	1 (4)
**Optic nerve, n (%)**	
* No alterations*	6 (24)
* Glaucomatous optic neuropathy*	18 (72)
* Optic atrophy*	1 (4)

CCT: central corneal thickness, CDVA: corrected distance visual acuity, CFC: cyclophotocoagulation, DALK: deep anterior lamellar keratoplasty, DMEK: Descemet membrane endothelial keratoplasty, DSAEK: Descemet stripping with automated endothelial keratoplasty, IOL: intraocular lens, IOP: intraocular pressure, IQR: interquartile range, SD: standard deviation.

**Table 2:** Changes in endothelium count, central corneal thickness, visual acuity, and intraocular pressure (n = 25 eyes).

Parameters *	Preoperative	6 Months	Last Follow-Up	*p* ^†^	*p* ^‡^
**ECD (cells/mm^2^)**	NA	1309 (994–2094.5) [630–2465]	1059 (750–1810) [556–2444]	-	0.052
**CCT** **(µm)**	739 (621–847) [547–1662]	595.5 (549.5–662.5) [468–759]	570 (531–604) [457–844]	**0.002**	0.330
**CDVA ^§^ (LogMAR)**	1.3 (1.3–1.3) [0.7–1.3]	1 (1–1) [0.4–1.3]	0.4 (0.25–0.5) [0.2–1]	**<0.001**	**<0.001**
**IOP** **(mmHg)**	12 (11–14) [9–31]	13 (12–16) [6–21.9]	13 (10–15) [5.1–25.5]	0.543	0.448

CDVA = corrected distance visual acuity, CCT = central corneal thickness, ECD = endothelial cell density, IOP = intraocular pressure, NA = data not available. Statistically significant values are in bold type. * Values reported as median (interquartile) [range]. ^†^ Wilcoxon’s signed-rank test for comparison between preoperative and 6-month data. ^‡^ Wilcoxon’s signed-rank test for comparison between 6-month and last control data. ^§^ For visual acuity analysis, only cases with potential visual function were included (n = 16).

## Data Availability

The datasets used during the current study are available from the corresponding author on reasonable request.

## Supplementary Materials

The following supporting information can be downloaded at: https://www.mdpi.com/article/10.3390/jcm13195715/s1, Video S1 showing the surgical steps.
